# Rapid Extraction of Lexical Tone Phonology in Chinese Characters: A Visual Mismatch Negativity Study

**DOI:** 10.1371/journal.pone.0056778

**Published:** 2013-02-20

**Authors:** Xiao-Dong Wang, A-Ping Liu, Yin-Yuan Wu, Peng Wang

**Affiliations:** 1 Division of Psychology, School of Humanities and Social Sciences, Nanyang Technological University, Singapore, Singapore; 2 Department of Economics, Anhui University, Hefei, China; 3 Department of Pediatric Oncology, Dana-Farber Cancer Institute and Children’s Hospital Boston, Harvard Medical School, Boston, Massachusetts, United States of America; 4 CAS Key Laboratory of Ion Beam Bioengineering, Institute of Technical Biology and Agriculture Engineering, Chinese Academy of Sciences, Hefei, China; Universität Bielefeld, Germany

## Abstract

**Background:**

In alphabetic languages, emerging evidence from behavioral and neuroimaging studies shows the rapid and automatic activation of phonological information in visual word recognition. In the mapping from orthography to phonology, unlike most alphabetic languages in which there is a natural correspondence between the visual and phonological forms, in logographic Chinese, the mapping between visual and phonological forms is rather arbitrary and depends on learning and experience. The issue of whether the phonological information is rapidly and automatically extracted in Chinese characters by the brain has not yet been thoroughly addressed.

**Methodology/Principal Findings:**

We continuously presented Chinese characters differing in orthography and meaning to adult native Mandarin Chinese speakers to construct a constant varying visual stream. In the stream, most stimuli were homophones of Chinese characters: The phonological features embedded in these visual characters were the same, including consonants, vowels and the lexical tone. Occasionally, the rule of phonology was randomly violated by characters whose phonological features differed in the lexical tone.

**Conclusions/Significance:**

We showed that the violation of the lexical tone phonology evoked an early, robust visual response, as revealed by whole-head electrical recordings of the visual mismatch negativity (vMMN), indicating the rapid extraction of phonological information embedded in Chinese characters. Source analysis revealed that the vMMN was involved in neural activations of the visual cortex, suggesting that the visual sensory memory is sensitive to phonological information embedded in visual words at an early processing stage.

## Introduction

In psycholinguistic research, the role of phonology in visual word recognition has long been an important issue. Recently, a considerable amount of research evidence supports the idea of an early and automatic activation of phonological encoding in visual word recognition [1], [2], [3], [4]. This literature is mostly based on the research findings in alphabetic scripts. However, empirical evidence is sparse in non-alphabetic scripts such as logographic Chinese, because logographic Chinese is unique in its design principle, including its phonological structure and the mapping and correspondence between orthographic and phonological representations. In the phonological structure, Chinese is the most spoken tonal language in the world, which uses lexical tones to differentiate word meaning, besides consonants and vowels [5], [6]. Moreover, the syllabic structure of Chinese differs largely from alphabetic languages: The smallest unit of meaning in Chinese is the mono-syllabic character [7]. In the mapping and correspondence between orthographic and phonological representations, unlike the alphabetic languages in which there is an intimate correspondence between the visual and phonological forms, there is no letter to sound correspondence in logographic Chinese [8]. The mapping between visual and phonological forms is relatively arbitrary in Chinese: One spoken syllable is often associated with multiple written characters (a one-to-many manner) and in Chinese, homophones are extensively used.

The neural mechanisms of rapid phonology encoding in alphabetic languages have been investigated using behavioral [9], [10], [11], [12], [13] and neuroimaging [14], [15], [16], [17], [18] methods. In a study investigating the automatic orthographic and phonological activation in the brief identification paradigm, Booth et al., found orthographic and phonological priming effects that favored the automatic activation of phonological information in visual words even in beginning readers [9]. It has also been demonstrated that word meaning is accessed through the automatic activation of phonological information [11]. The proposal of automatic activation of phonological information in visual word recognition has also been supported by evidence from neuroimaging studies. In an fMRI study, subjects were asked to perform a lexical decision task on prime-target pairs including word-word homophone and pseudoword-word pseudohomophone pairs with a prime presentation below perceptual threshold. The results revealed that several cortical regions exhibited hemodynamic response suppression due to phonological priming including bilateral superior temporal gyri (STG), middle temporal gyri (MTG), angular gyri (AG), and left lateralized supramarginal gyrus (SMG). This showed the automatic and implicit stages of phonological processing [18]. In high temporal resolution event-related potentials (ERP) studies on phonological processing in visual words, the majority of research however, focused on the N400 or even later stage components [19], [20], [21], [22].

Since the design principles of logographic Chinese and alphabetic languages are totally different, an interesting question is whether or not the phonology information is rapidly and automatically extracted in logographic Chinese. To address this issue, an effective approach is to use a technique with a sufficient temporal resolution to isolate the brain response component contributed by the cognitive processing at an early processing stage. To this end, the visual mismatch negativity (vMMN) can be an efficient tool for investigating early and automatic response to visual speech stimuli. The vMMN is suggested to be a visual equivalent of the auditory mismatch negativity (MMN) [23] and is an index of early and automatic encoding of the change of regularities in the visual modality [24]. It has been reported that vMMN can be elicited by deviants differing in spatial frequency [25], color [26], [27], line orientation [28], shape [29], [30], [31] and even abstract sequential regularities in a visual stimuli stream [32], [33]. It has been verified a memory-based change detection mechanism which underlies the vMMN and the vMMN is suggested to be a useful tool to study the function of visual sensory memory [34].

To address the issue of whether or not phonological information is rapidly and automatically extracted from the Chinese characters, an important aspect is embedding an implicit phonological commonness in a varying visual word stream. To this end, Chinese homophones are ideal visual materials. In contrast to alphabetic orthographies, Chinese characters have arbitrary mappings between orthographic and sound forms, and a unique property of Chinese homophones is that they differ in orthography and meaning but have the same phonological information.

**Figure 1:**
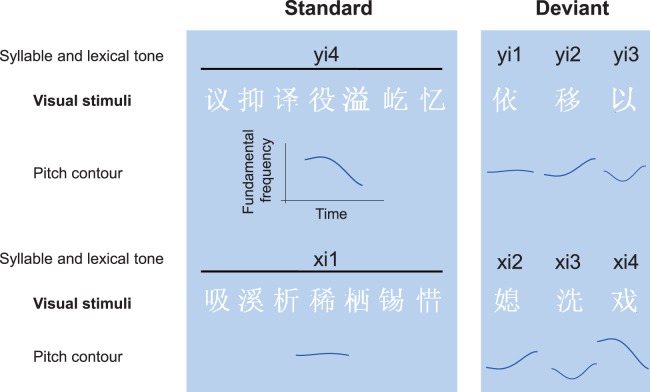
Visual characters used for stimuli. Standard stimuli are homophones of Chinese characters pronounced as “yi4” (or “xi1”), deviant stimuli are characters pronounced as “yi1”, “yi2” and “yi3” (or “xi2”, “xi3” and “xi4”). The differences between the standards and deviants are implicit auditory phonology, in this case the pitch contours of lexical tones.

**Figure 2:**
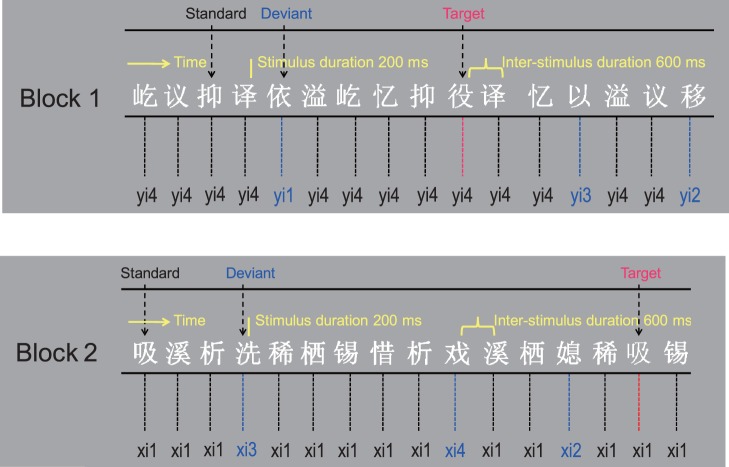
Experimental design and samples of the varying visual stream constructed by Chinese characters. The subjects’ task was a rapid button-press to the changes of color of the characters. Compared to the standard stimuli, half of target stimuli differed only in color and half differed in both color as well as the implicit auditory phonology.

In the present study, we continuously presented Chinese characters differing in orthography and meaning to native Mandarin Chinese speakers to construct a constant varying visual stream. In the stream, most stimuli were homophones of Chinese characters: The phonological features embedded in these homophones were the same, including consonants, vowels and the lexical tone (Fig. 1). These homophones were defined as the standard stimuli. Occasionally, this implicit phonological commonness was randomly violated by characters whose phonological features differed in the lexical tone. These characters were defined as the deviant stimuli (Fig. 2). The extraction of the implicit phonological information in the visual word stream at an early processing stage was measured using whole-head recordings of the vMMN. During the recording, subjects were asked to detect black colored characters and make a button press as quickly and accurately as possible.

## Materials and Methods

### Subjects

Fourteen native speakers of Mandarin Chinese (8 females) with no history of neurological or psychiatric impairment participated in the present study. The subjects were 21–28 years old, musically untrained, and right-handed according to an assessment with the Chinese version of the Edinburgh Handedness Inventory [35]. All subjects were advanced adult native Mandarin Chinese speakers with good reading and writing skills. Nine of them (6 females) also participated in a following supplementary experiment. They were compensated for their participation. The experimental protocol was approved by the institutional review board of the Institute of Technical Biology and Agriculture Engineering of Chinese Academy of Sciences. All participants provided written informed consent after the nature of the experiment had been fully explained. All participants had normal or corrected-to-normal vision.

### Stimuli and Procedure

All visual words used in this study were Chinese characters and were matched for average stroke numbers across the standard and deviant conditions. In addition, the homophone occurrence frequency was matched between the standard and deviant conditions since it might exert an influence on the phonological activation in Chinese word identification. In the varying visual stream of Chinese characters, most characters are homophones and share the same phonological information including consonants, vowels and the lexical tone to form an implicit phonological commonness (all these characters pronounced “yi4” (or “xi1”)) (Fig. 1). Occasionally, this phonological commonness was violated by some other characters with different tones “yi1”, “yi2” and “yi3” (or “xi2”, “xi3” and “xi4”). All characters were presented in the odd-ball paradigm, the standard stimuli were homophones that pronounced “yi4” in block 1 and “xi1” in block 2, and the deviant stimuli were characters that pronounced “yi1”, “yi2” and “yi3” in block 1 and “xi2”, “xi3” and “xi4” in block 2 (Fig. 2). The visual word stimuli subtending 2 visual degrees were presented on a computer monitor in a middle gray background at a viewing distance of 50 cm. Within each block, the standard stimuli were presented with a probability of 70%, deviant stimuli 10% and the target stimuli 20%. Stimuli were pseudo-randomly presented for 200 ms, followed by a 600 ms interstimulus interval. Two deviants or targets never appeared in immediate succession. Between two infrequent stimuli, there were at least three standard stimuli. The standard and deviant stimuli were presented in a white color, the target stimuli were presented in a black color. Participants were instructed to detect black colored characters by pressing a button as quickly and accurately as possible. In each block, a total of 700 stimuli were presented. Each block was presented twice and block order was fully counterbalanced between participants. We also ran a supplementary experiment with the standard and deviant stimuli presented in two separate blocks. In one block, the standard stimuli were presented with a probability of 80% and target stimuli 20%. In the other block, the deviant stimuli were presented with a probability of 80% and target stimuli 20%.

### Data Recording and Analysis

Electroencephalogram (EEG) was recorded and filtered on-line with a low-pass of 100 Hz and sampled at a rate of 500 Hz. An electrode cap carrying 66 Ag/AgCl electrodes placed at standard locations covering the whole scalp was used (the extended international 10–20 system). The reference electrode was attached to the tip of the nose, and the ground electrode was placed on the forehead. Electrode impedances were kept <5 k Ohm. The recording data were filtered off-line between 1 and 25 Hz (24 dB/octave) with a finite impulse response filter. Epochs of 800 ms time window, starting 100 ms before the onset of stimulus were obtained from the continuous data and rejected when fluctuations in potential values exceeded ±75 µV. The ERPs evoked by standard and deviant stimuli were calculated by averaging individual trials (excluding standards that immediately followed a deviant or a target). vMMN was derived by subtracting the ERP response to the standard from that to the deviant stimuli. Parieto-occipital and fronto-central regions over the scalp were chosen to be the regions of interest. The vMMN was maximal over the parieto-occipital scalp and analyzed at electrodes OZ, O1, O2, CB1, CB2, PO7, PO8, PO5, PO6, PO3, PO4, P7, P5, P3, P1, PZ, P2, P4, P6 and P8. For the fronto-central region, electrodes F3, F1, FZ, F2, F4, FC3, FC1, FCZ, FC2, FC4, C3, C1, CZ, C2 and C4 were analyzed. The same methods were also used to analyze the data of the supplementary experiment, in which the standard and deviant stimuli were presented in separate blocks. To localize the neural source of the vMMN in response to the violation of the implicit phonological information embedded in the visual character stream, we evaluated the source of the vMMN using L2 minimum-norm current estimates (L2 MNE). The method of minimum norm supplies a solution to localize the neural activity inside the brain from the scalp EEG signals, and uncovers the aggregation of active neuronal current elements with the smallest amount of overall activity [36], [37]. Such source estimation does not require a priori assumptions about underlying neural generators and attempts to minimize the response activity that can account for the scalp potentials [37]. The MNE solution calculated for grand-average responses rather than individual data and hence reduced noise and improved signal-to-noise ratio. To focus the cortical source of the vMMN activity, we further conducted a source analysis solution using CLARA (Classical LORETA Analysis Recursively Applied), which is an iterative application of the LORETA algorithm with an implicit reduction of the source space in each iteration to make distributed source images more focal. The MNE and CLARA were done using the Besa software package (Megis Software, Munich, Germany).

## Results

### Electrophysiological and Behavioral Responses to the Target Stimuli

**Figure 3:**
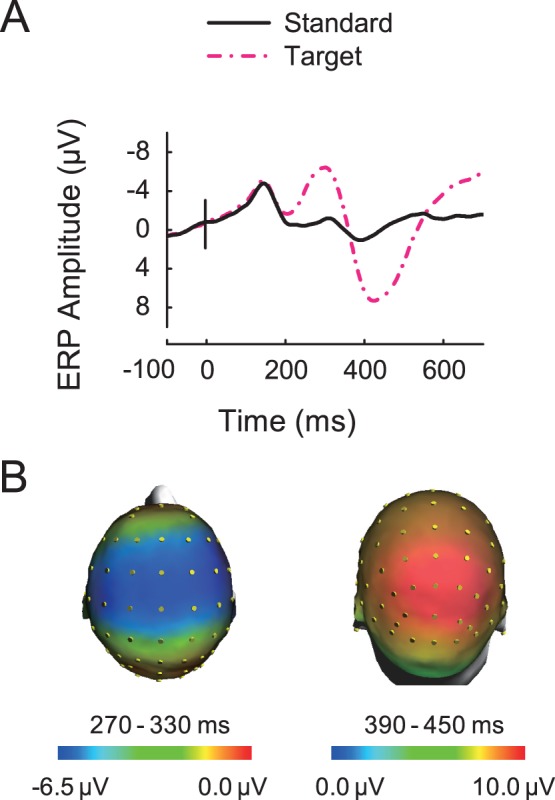
Grand average of event-related potentials (ERPs) in response to the standard and target stimuli. (*A*) Grand-averaged ERPs for standard (solid black line) stimuli and target (dash dotted red line) stimuli at the FCZ electrode. (*B*) Scalp topographic maps of N2 component (*Left*) and P3 component (*Right*) for target stimuli.

Fig. 3 displays the grand-averaged ERPs in response to the standard stimuli and target stimuli at the FCZ electrode. The target stimuli evoked robust N2 and P3 components (Fig. 3*A*). As shown in Fig. 3*B*, the N2 component was recorded with a central scalp distribution, and the P3 component was recorded with a parieto-occipital scalp distribution. Behavioral responses to the targets revealed a high proportion of hits (mean = 93±3.9% (s.e.m.)). The mean RT was 416±13.8 ms (s.e.m.).

### P1-N170 Complex Evoked by Standard Stimuli and their Lateralized Effects

**Figure 4:**
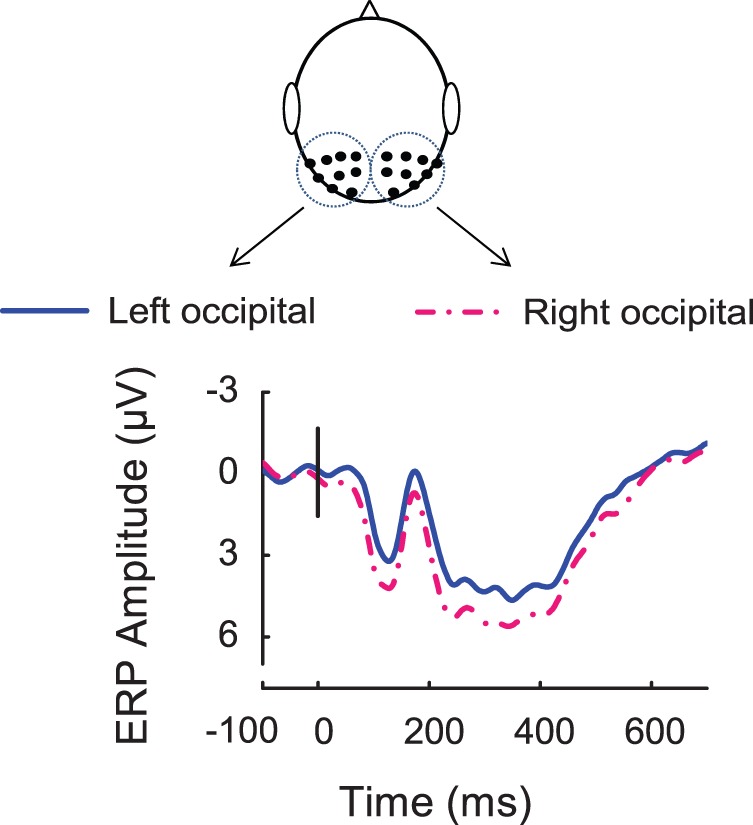
P1-N170 complex evoked by standard stimuli. ERPs in response to the standard stimuli showed maximal P1-N170 responses over parieto-occipital scalp areas in the left and right hemispheres. P1-N170 complex in the left hemisphere (solid blue line, linear derivation of electrodes P7, P5, P3, P1, PO7, PO5, PO3, CB1, and O1), P1-N170 complex in the right hemisphere (dash dotted red line, linear derivation of electrodes P8, P6, P4, P2, PO8, PO6, PO4, CB2, and O2).

Standard stimuli evoked a robust P1-N170 complex. As shown in Fig. 4, electrodes in the parietal-occipital region were chosen for analysis because the amplitudes of P1-N170 complex were maximal. The amplitudes of P1 component were significantly larger in the right hemisphere than in the left [F (1, 13) = 6.3, P<0.05], while for the N170 component, the amplitudes in the left hemisphere were significantly larger than those in the right hemisphere [F (1, 13) = 7.17, P<0.05].

### Violation of Implicit Phonological Information Evoked a Robust vMMN Response

**Figure 5:**
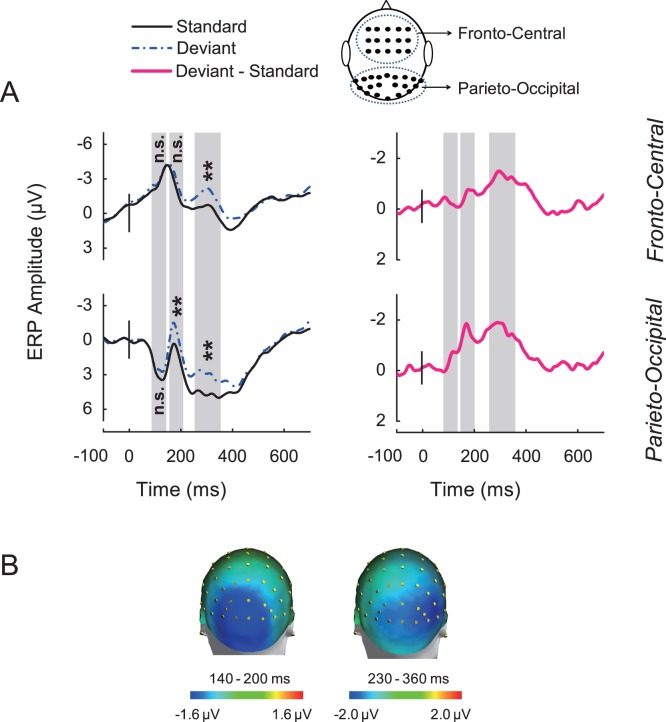
Grand average waveforms and topographical maps of visual mismatch negativity. (*A*) ERPs elicited by the standard and deviant stimuli (*Left*) and amplitude difference between ERPs elicited by deviants and standards (*Right*) over the fronto-central and parieto-occipital scalp areas. (*B*) Scalp topographic maps of vMMN in the 140–200 ms (*Left*) and 230–360 ms (*Right*) time windows.

**Figure 6:**
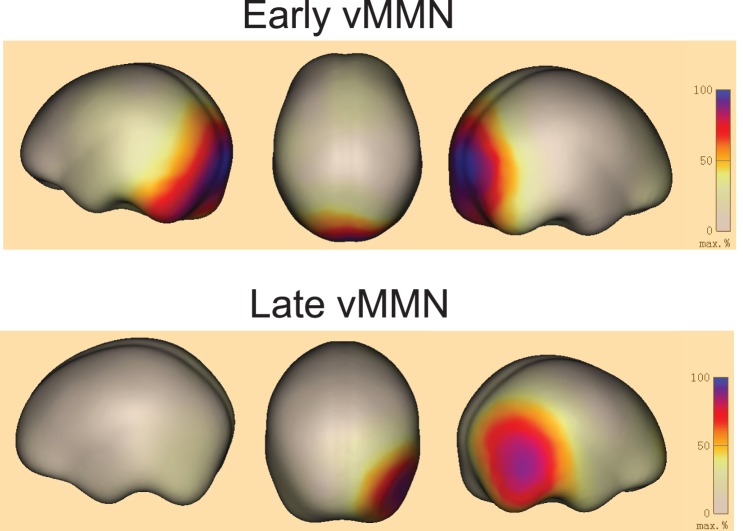
Grand average localization of vMMN generators. The minimum-norm estimate of the sources of the early vMMN (*upper panel*) and late vMMN (*lower panel*) at their peak latencies.

**Figure 7:**
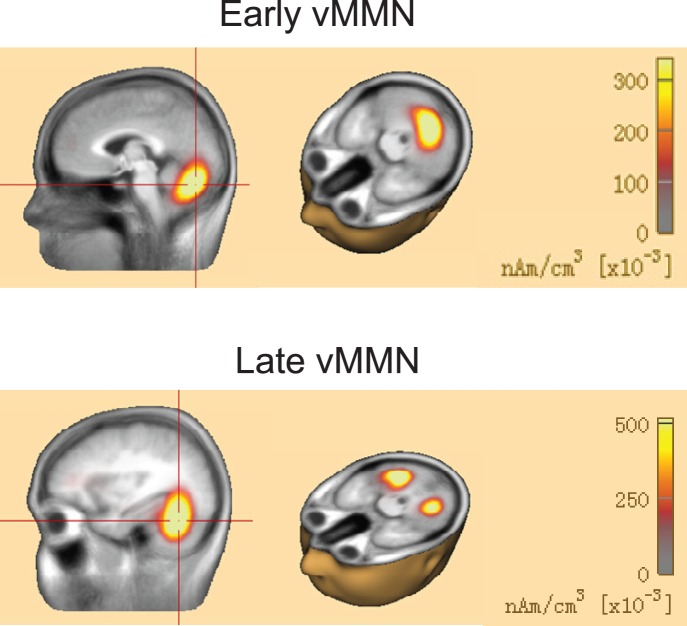
Grand average localization of vMMN generators estimated by CLARA (Classical LORETA Analysis Recursively Applied). CLARA is an iterative application of the LORETA algorithm to make distributed source images more focal. Source localization for the early vMMN (*upper panel*) and late vMMN (*lower panel*) at their peak latencies.

To evaluate the vMMN, electrodes in the fronto-central and parietal-occipital regions were chosen for statistics. ERP amplitudes elicited by standard stimuli and deviant stimuli were calculated in three time windows: 80–130 ms, 140–200 ms and 230–360 ms. In the parietal-occipital region, ERPs in response to the deviant stimuli and standard stimuli differed significantly in the 140–200 ms and 230–360 ms time windows [F(1, 13) = 11.46, P<0.01 and F(1, 13) = 17.82, P<0.01 respectively]. In the fronto-central region, ERP in response to the deviant stimuli and standard stimuli differed only significantly in the 230–360 ms time window [F(1, 13) = 13.44, P<0.01] (Fig. 5*A* *Left*). The vMMN was derived by subtracting the ERP response to the standard from that to the deviant stimuli (Fig. 5*A* *Right*). The scalp topographic maps of vMMN responses in the 140–200 ms and 230–360 ms time windows showed both parieto-occipital distributions (Fig. 5*B*). To further confirm that the vMMN we recorded was evoked by the violation of the lexical tone phonology rather than the differences of orthographies or semantics between the standard and deviant stimuli, we presented subjects with standard stimuli and deviant stimuli in separate blocks. Results showed that the ERPs evoked by the two sets of stimuli did not differ significantly in the parietal-occipital region or the fronto-central region in either time window (Fig. S1): For the parietal-occipital region, [F(1, 8) = 0.02096, P = 0.88846], [F(1, 8) = 0.43971, P = 0.5259] and [F(1, 8) = 0.02649, P = 0.87474] for the 80–130 ms, 140–200 ms and 230–360 ms time windows, for the fronto-central region, [F(1, 8) = 0.38257, P = 0.55343], [F(1, 8) = 0.0669, P = 0.80244] and [F(1, 8) = 0.36374, P = 0.56314] for the 80–130 ms, 140–200 ms and 230–360 ms time windows. The cortical sources for the vMMNs in the early time window (early vMMN) and late time window (late vMMN) estimated using L2 MNE demonstrated that the vMMNs were involved in neural activations of the visual cortex (Fig. 6). The proposal of visual cortex responds to lexical tone phonology received further support from the source analysis using CLARA, an iterative application of the LORETA algorithm with an implicit reduction of the source space in each iteration to make distributed source images more focal (Fig. 7).

## Discussion

This study examined the rapid extraction of phonological information in the context of visual words presentation at an early stage of visual processing using vMMN, an electrophysiological index of early and automatic deviant detection in the visual modality. By virtue of the unique properties of homophones of Chinese characters, we constructed a constant changing visual stream of Chinese characters with an implicit phonological commonness embedded in it. This commonness was occasionally violated by characters whose phonology differed in the lexical tone. The robust vMMN evoked by the deviant visual character stimuli suggests that the lexical tone phonology is processed rapidly and automatically in visual sensory memory. Source analysis revealed that the vMMN was involved in neural activations of the visual cortex. Our results indicate that the visual sensory memory has already been sensitive to the phonological information embedded in the visual word stream at an early processing stage and this cross-model effect in the native Mandarin Chinese speakers is due to the activation of long-term memory of the lexical tone information embedded in the characters.

Previous research using ERP suggested that phonological processing in the visual word stream occurs rather late and is task relevant [19], [20], [21], [22]. Our results verified the early stage processing of the phonological information in the context of visual word stream when the subjects performed a task which was irrelevant to the phonological, lexical or semantic processing. Learning the correspondence between a speech sound and its written form is a prerequisite for the development of reading and writing skills [38]. It is believed that deficient written and speech association forms the basis of difficulties in learning to read in developmental dyslexia [39], [40]. Since the subjects recruited in this study were advanced adult native Mandarin Chinese speakers with good reading and writing skills, the recorded robust vMMN indicates the activation of the long-term memory of the phonological information embedded in the visual word: In this case the lexical tones. Studies also showed that the vMMN reflects a memory-based change detection neural substrate [34], and is consistent with the neural substrate of the auditory MMN [41]. In the auditory domain, numerous studies revealed the effects of long-term memory and experience on the early auditory processing of speech sounds, as revealed by the auditory MMN [41], [42], [43], [44], [45]. Our results indicate that this is also true in the visual domain, as revealed by the vMMN.

In this study, the visual character stimuli evoked a robust P1-N170 complex. Analysis of peak latencies and signal amplitudes of the P1 component from the left and right parieto-occipital scalp regions revealed that the P1 ERP response, traditionally associated with low-level perceptual processing [46], was lateralized to the right brain hemisphere (Fig. 4), and is consistent with the proposal that the right hemisphere preferentially processes tasks with patterns and specific shape information [47], and word form information [48], [49]. With respect to the N170 component, the response amplitude was significantly larger in the left occipital region than in the right (Fig. 4), reflecting the neural change resulting from extensive experience with the particular type of visual category [50]. The left lateralized N170 response recorded in this study is in line with the current literature which suggested that in skilled readers, the N170 response to visual words is typically left lateralized, and is contrary to the right lateralized or bilateral N170 response to faces or objects [51], [52], [53], [54].

Lexical tones, auditory pitch patterns in voice fundamental frequency, are used to signal word meaning in tonal languages. For the cognitive processing of lexical tones, current literature exclusively focuses on the auditory modality. For instance, evidence from neural imaging studies such as fMRI and PET demonstrated that auditory processing of lexical tones engaged a functional dependent mechanism and hence lateralized to the left brain hemisphere [55], [56], [57]. Recent evidence from EEG studies demonstrated that auditory processing of lexical tones at an early, pre-attentive stage was lateralized to the right brain hemisphere [58], [59], which supports an acoustic dependent mechanism for the early auditory processing of lexical tones [60]. As the auditory MMN is sensitive to the acoustic properties of speech sound, it is very difficult to tease apart pure acoustic effects of lexical tones (change of the pitch contour) from those of linguistic origin (signal a word meaning). Thus, it is still possible that there is a long-term memory effect on the early auditory processing of lexical tones, but this effect may be overwhelmed by the salient acoustic properties of pitch patterns of lexical tones, and the right lateralized MMN responses to the lexical tone contrast may not rule out the potential effects of the long-term memory in the native speakers [61]. In the present study, by virtue of Chinese homophones and vMMN, we ruled out confounds of the acoustic properties of lexical tones and investigated the native speakers’ long-term memory for lexical tones in the context of visual word processing. Our results verified the existence of long-term memory effect of lexical tones in the natives. Surprisingly, this cross-model effect based on the long-term memory of lexical tones arose as early as 170 ms after the onset of the visual characters, indicating an early memory-dependent phonological activation in visual words.

It is interesting and counterintuitive that sensory-specific cortices appear to be sensitive to information from another modality [62], [63], [64]. Letters and speech sound processing, a typical example of audio and visual information integration is extensively investigated recently and it has been shown that audiovisual speech processing involves multisensory integration regions including low level auditory and visual sensory systems [65], [66], [67]. Learning the culturally defined associations between letters and speech sounds forms the basis of reading in alphabetic scripts and is a prerequisite for the development of reading and writing skills [38]. In the present study, source analysis showed that the vMMN was involved in neural activations of the visual cortex (Fig. 6 and 7), suggesting that the visual sensory memory is sensitive to auditory phonological information embedded in visual words at an early processing stage. Since the auditory processing of speech sounds is modulated by visual letters [68], our results provide evidence for the opposite direction, the processing of visual words was influenced by the auditory phonology embedded in the visual characters even at an early stage of visual processing.

The observation of the rapid brain response to phonological information as shown in the present study may be relevant to educational and clinical considerations. In recent years, the number of tonal language speakers is increasing even in the nations of non-tonal languages. Learning the correspondences between the Chinese characters and speech sounds forms the basis of the development of reading and writing skills in learning Chinese. A vast variety of hearing, speech, and language disorders can weaken the capacity to perceive or produce the lexical tone, consequently impairing the communicative abilities of people [69]. The auditory MMN is suggested to be a potential tool in clinical and other applications [23], [70], [71]. Similarly, the vMMN is also considered to be a potential tool in cognitive dysfunction [72], [73] and learning [32]. In this sense, the experimental procedure deployed in the present study may be applied to test the effect of learning the correspondences between the written forms and speech sounds, which is a prerequisite for the development of reading and writing skills [38].

In this study, we used Chinese characters in a modified visual odd-ball paradigm. This paradigm was adopted from several vMMN studies in which the visual stimuli were presented in subjects’ visual field while the deviant stimuli were task-irrelevant [74], [75], [76]. It should be noted that in the present study and those vMMN studies, the visual stimuli were not fully presented outside the focus of subjects’ attention, and it was still possible for subjects to have expectancy for the repetition of the visual stimuli. In this case subjects might be aware of the deviant stimuli that violated their expectancies. With respect to the present study, the recorded vMMN which indexed an early stage visual processing may not be fully automatic because of subjects’ possible expectancy for the repetition of the stimuli. The task for the subjects is to detect characters with a different color. Although the task is not relevant to lexical tone processing, subjects’ expectancy for the repetition may have an effect on the lexical tone extraction. A potential solution is to present the visual stimuli at the peripheral areas rather than at the center of the fovea, as used in some recent vMMN studies [33], [77]. A fully verification of the automation of vMMN needs further investigation and improved paradigms.

In summary, we addressed the issue of whether or not phonological information is rapidly and automatically extracted in Chinese characters by the brain, and we showed that the violation of the lexical tone phonology evoked an early, robust visual response, as revealed by whole-head electrical recordings of the vMMN. This indicates the rapid extraction of phonological information embedded in visual characters. Our results further suggest the activation of long-term memory effect of the lexical tone phonology embedded in visual characters and will help to understand the neural mechanisms underlying our remarkable capacity of visual cortex in the processing of phonological information.

## Supporting Information

Figure S1 **Event-related potentials (ERPs) in response to standard and deviant stimuli presented in separate blocks.** ERP responses over the fronto-central sites (*upper panel*) and over the parieto-occipital sites (*lower panel*).
(EPS)
